# Complete chloroplast genomes of *Impatiens cyanantha* and *Impatiens monticola*: Insights into genome structures, mutational hotspots, comparative and phylogenetic analysis with its congeneric species

**DOI:** 10.1371/journal.pone.0248182

**Published:** 2021-04-02

**Authors:** Chao Luo, Yang Li, Roshani Budhathoki, Jiyuan Shi, Huseyin Yer, Xinyi Li, Bo Yan, Qiong Wang, Yonghui Wen, Meijuan Huang, Haiquan Huang

**Affiliations:** 1 College of Landscape Architecture and Horticultural Science, Southwest Forestry University, Kunming, China; 2 Research and Development Center of Landscape Plants and Horticulture Flowers, Southwest Forestry University, Kunming, China; 3 Yunnan Province Engineering Research Center for Functional Flower Resources and Industrialization, Southwest Forestry University, Kunming, China; 4 Department of Landscape Architecture and Plant Science, University of Connecticut, Storrs, CT, United States of America; National Agri-Food Biotechnology Institute (NABI) Mohali, INDIA

## Abstract

*Impatiens* L., the largest genus in the family Balsaminaceae with approximately 1000 species, is a controversial and complex genus that includes many economically important species well known for medicinal and ornamental values. However, there is limited knowledge of molecular phylogeny and chloroplast genomics, and uncertainties still exist at a taxonomic level. In this study, we have assembled four chloroplast genomics specimens of *Impatiens cyanantha* and *Impatiens monticola*, which are found at the different altitudes of Guizhou and Yunnan in China, and compared them with previously published three wild Balsaminaceae species (*Impatiens piufanensis*, *Impatiens glandlifera*, and *Hydrocera triflora*). The complete chloroplast genome sequences ranged from 152,236 bp (*I*. *piufanensis*) to 154,189 bp (*H*. *triflora*) and encoded 115 total distinct genes, of which 81 were protein-coding, 30 were distinct transfer RNA genes(tRNA), and 4 were ribosomal RNA genes (rRNA). A comparative analysis of *I*. *cyanantha* (Guizhou) vs. *I*. *cyanantha* (Yunnan) and *I*. *monticola* (Guizhou) vs. *I*. *monticola* (Yunnan) revealed minor changes in lengths; however, similar gene contents, gene orders, and GC contents existed among them. Interestingly, highly coding and non-coding genes, and regions *matK*, *psbK*, *atpH-atpI*, *trnC-trnT*, *petN*, *psbM*, *atpE*, *rbcL*, *accD*, *psaL*, *rps3-rps19*, *ndhG-ndhA*,*rpl16*, *rpoB*, *ndhB*, *ndhF*, *ycf1*, and *ndhH* were found, which could be suitable for identification of species and phylogenetic studies. During the comparison between *I*. *cyanantha* (Guizhou) and *I*. *cyanantha* (Yunnan), we observed that the *rps4*, *ycf2*, *ndhF*, *ycf1*, and *rpoC2* genes underwent positive selection. Meanwhile, in the comparative study of *I*. *monticola* (Guizhou) vs. *I*. *monticola* (Yunnan), The *accD* and *ycf1* genes were positively selected. Additionally, phylogenetic relationships based on maximum likelihood (ML) and Bayesian inference (BI) among whole chloroplast genomes showed that a sister relationship with *I*. *monticola* (Guizhou) and *I*. *monticola* (Yunnan) formed a clade with *I*.*piufanensis* proving their close connection. Besides, *I*.*cyanantha* (Guizhou) and *I*. *cyanantha* (Yunnan) formed a clade with *I*. *glandlifera*. Along with the findings and the results, the current study might provide valuable significant genomic resources for systematics and evolution of the genus *impatiens* in different altitudes of regions.

## Introduction

*Impatiens* L., which belong to the family Balsaminaceae, have been well known and used as medicinal, horticultural, and ornamental plants in North America, Europe, and China [1]. The family of Balsaminaceae consists of only two genera of *Impatiens* and the monospecific sister species *Hydrocera triflora*, with strong similarity in morphology and genomics DNA datasets [2]. About 1000 species are involved in *Impatiens*, distributing from tropics to subtropics, and extending from sea level to an altitude of 4,000 meters [3]. *Impatiens* live in roadside ditches, valleys, mesic or wet areas, and by the streams or even in much drier habitats [4]. Tropical Africa, Madagascar, Sri Lanka, Himalayas, and Southeast Asia are the biodiversity hotspots for the endemic Balsaminaceae [5]. Southwest China is the original biodiversity and distribution center of the Balsaminaceae species [6, 7].

In ancient China, *Impatiens* were called ’zhijiahua’, which were used for crushing into mashes and directly applied on the nails [8, 9]. They are also considered as annual herbs for the medical treatment of rheumatism, beriberi, bruises, pain, wart, snakebite, fingernail inflammation, and onychomycosis [10, 11]. A broad range of pharmaceutical and chemical products such as fatty acids, naphthoquinones, phenolic acids, flavonoids, anthocyanidins, peptides, and saponins have recently been characterized in this plant [12]. Additionally, previous research has demonstrated that the *Impatiens* species can accumulate high levels of metals such as copper, zinc, chromium, and nickel. Moreover, it has a strong phytoremediation potential of soils heavily polluted by cadmium and copper [13, 14].

*Impatiens* are known to flower diversely and be morphologically variable [15]. The genus is characterized by zygomorphic flowers with enormous diversity and high levels of convergent evolution variability in corolla color and morphology. The flowers are incredibly fragile, and most are coalesced and folded in dried specimens that makes it difficult to separate and reconstruct different parts [16]. Early research on *Impatiens* was primarily focused on a specific geographical area that provided purely descriptive traditional taxonomy [17]. Up to now, based on several plastids (such as coding genes *matK*, *rbcL*, *trnK*, and intergenic regions *atpB-rbcL* and *trnL-trnF*), the infrageneric molecular classification for *Impatiens* was obtained [18, 19]. However, the existing published data contained only a few samples from prominent regional characteristics [20]. Some species (for example, *Impatiens monticola* and *Impatiens cyanantha*) with diversified morphology have taxonomic controversy due to unresolved phylogenetic relationships.

Temperature and rainfall have a direct impact on the growth and development of plants [21]. It was reported that altitude might affect the same plant in a different manner [22]. The difference in altitude can determine the leaf morphological traits. The thicker leaves are observed more in the high-altitude plants than in those of low-altitude [23]. Thus, the variation of leaf traits based on the altitudinal patterns is probably associated with plant ecotypes and phenotypic plasticity, and maybe some links between altitude and plant morphology [24]. The plants must deal with multiple environmental factors such as temperatures, air humidity, UV radiation, atmospheric pressure with the variation of altitudinal gradients [25]. In response to climatic variations, the plants’ physiological processes and phenotypic traits should be regulated and modified. Chloroplasts are the primary sources of reactive oxygen species in plants [26]. Thus, an accurate estimate of the genetic variation along with the altitude gradient is essential for the conservation and sustainable use. Therefore, using whole chloroplast genomes as the evolution analysis is urgently needed, improving the understanding of the phylogenetic relationships and contributing to molecular plant breeding.

In the present study, by using Illumina sequencing technology, we assembled four chloroplast genomics specimens of *Impatiens cyanantha* and *Impatiens monticola*, which are located at different altitudes from Guizhou and Yunnan in China [27]. The present investigation is a novel attempt to reveal and identify the phylogenetic analysis of the taxonomic position of *Impatiens* based on the whole chloroplast genome. The aims of this study are: (i) to conduct a comprehensive research of the pomegranate chloroplast genome, including basic chloroplast genome structure information, codon usage, repetitive structure characteristics, inverted repeat (IR) region expansion, contraction, and comparative genomic divergence; (ii) to further understand the relationships of the *Impatiens* species; and (iii) to reconstruct and analyze the phylogenetic tree based on the complete chloroplast genomes. This study will contribute to future research on phylogeny, taxonomy, population genetics, genetic engineering studies of *Impatiens* species. Finally, it will also provide critical information for the systematics and evolution of *Impatiens*.

## Materials and methods

### Ethical statement

No specific permits were required for the collection of specimens for this study. This research was carried out in compliance with the relevant laws of China.

### Sampling and DNA extraction

Leaf samples from plants were collected from different locations, and the samples were deposited in the plant laboratory of the College of Landscape architecture and Horticulture Science, Southwest Forestry University, Kunming, Yunnan, China (Table 1). Fresh leaves were collected and immediately stored in the liquid nitrogen [28]. We extracted the genomic DNA by using the Tiangen DNA Reagent Extraction Kit [29]. And 5–10 μg of genomic DNA quality was checked using spectrophotometry [30].

**Table 1:** The list of basic information of *Impatiens* specimens.

Specimens	Altitude	Latitude and Longitude	Location	Voucher Specimen
*I*. *monticola* (Guizhou*)*	818m	N28°9′56″	Suiyang Kuankuoshui Nature Reserve, Zunyi City, Guizhou Province, China	SWFU-IBSD20180823
		E107°12′34″		
*I*.*monticola* (Yunnan)	1220m	N23°13′285″	Malipo Laoshan Nature Reserve, Wenshan City, Yunnan Province, China	SWFU-IBSD20180910
		E104°85′667″		
*I*. *cyanantha* (Guizhou)	1777m	N24°07′296″	Chahe Village, Pingdi Township, Pan County, Liupanshui, Guizhou Province, China	SWFU-IBLH20180817
		E104°07′614″		
*I*. *cyanantha* (Yunnan)	3158m	N25°58′20″	Jizu Mountain Scenic Area, Dali City, Yunnan Province, China	SWFU-IBLH20180920
		E100°21′35″		

### Illumina sequencing, assembly, and annotation

First, the samples were sequenced on an Illumina HiSeqX instrument (Biozeron, Shanghai, China). Approximately 2 G raw data were generated with read lengths of 150 bp, and the chloroplast genome sequencing depth was nearly 60×. Next, the quality of paired-end Illumina reads was assessed in FastQC, and the pipeline GetOrganelle version 1.6.2. was used to select trimmed reads with default settings that corresponded to the plastid using the plastome of *I*. *piufanensis* as a reference [31]. Finally, the plastid filtered reads from GetOrganelle version 1.6.2 were imported in Geneious R8.0.2 with default settings. The de novo assembly was conducted with Velvet implemented in Geneious with the K-mer ranging from 69 to 99. The best K-mer was determined with the Velvet Optimiser implemented in Geneious with the K-mer choice. The predicted annotation of each assembled chloroplast genome was performed by the online program DOGAM (Dual Organellar Genome Annotator) with default values or the GeSeq (version 1) using the default parameters to predict protein-coding genes by HMMER profile search and ARAGORN v1.2.38. Then, the start and stop codon positions were further analyzed by the homologous gene identification [32]. Besides, the position of tRNA was confirmed with tRNAscan v1.23 [33]. The intron and exon boundaries of protein-coding genes were manually corrected, when necessary, and verified using Geneious R8.0.2 by realigning with references [34]. The physical chloroplast genome maps were generated by the Chloroplot software with the default setting and checked manually [35].

### Analysis of tandem repeats and single sequence repeats

The Geneious R8.0.2 software was utilized to calculate the GC content. Online MISA software was considered to detect SSRs with the minimal repeat numbers set to 10, 5, 4, 4, 4, and 4 for mono-, di-, tri-, tetra-, penta-, and hexanucleotides, respectively [36]. The REPuter identified the size and location of forward, reverse, complement, and palindromic repeat sequences [37]. The followings are the settings parameters: (1) Hamming distance is equal to 3; (2) minimal repeat size, 30 bp; and (3) maximum computed repeats, 90 bp. The software CodonW (1.4.4) was implemented for investigating the distribution of codon usage, which was analyzed with the relative synonymous codon usage (RSCU) ratio [38].

### Chloroplast genome alignment

To detect the divergence hotspots, the online software MAFFT was selected to align the whole chloroplast genomes [39]. The whole-genome alignment of impatiens and other species was compared by mVISTA in Shuffle-LAGAN mode and using the *I*. *piufanensis* genome as a reference to detect possible gene losses, gene variation, or gene conservation [37]. DnaSP v5.10 was operated to calculate the nucleotide divergence values using the sliding window method with a window length of 800 bp and a 200 bp step size [40]. Genome-Wide omparison was aligned with the *H*. *triflora* chloroplast genome, using the MAUVE v.2.4.0 software with a default "seed families" and default values for all other parameters, and then was concatenated using MAFFT program v7.309 in Geneious [41].

### Adaptive evolution analysis

To evaluate the evolutionary rate variation, the nonsynonymous (dN) and synonymous (dS) substitution rates and their ratio (ω = dN/dS) were analyzed. The same protein-coding regions were extracted using Geneious R8.0.2 software. Gaps and stop codons were manually removed, and the sequences were separately aligned using MAFFT. The aligned files were converted into AXT format using the parse Fasta Into AXT.pl Perl script^2^. The values of dN, dS, and dN/dS for each gene were calculated with the software, KaKs_calculator 1.2, using the default model.

### Phylogenetic analyses

Based on the complete chloroplast genomes, we used the phylogenetic tree to explore the phylogenetic positions and evolutionary relationships of *I*. *cyanantha* and *I*. *monticola* species. These chloroplast genomes from seven families within impatiens included seven Balsaminaceae specimens, six Primulace species, five Ebenace species, four Theace species, two Saxifragace species, four Actinidiace species, and one Styracace species as outgroups. The aligned sequences were concatenated by MAFFT version 7.222 [42]. The Maximum likelihood (ML) and Bayesian Inference (BI) were conducted for the topologies. The ML analysis was implemented in RAxML v.8.2.9 [43] and IQ-TREE ver. 1.6.1 [44]. Based on the Akaike information criterion (AIC), the best fitting was GTR+F+I+G4 substitution model with 1000 bootstrap replicates for ML analyses [45]. The Bayesian inference (BI) tree was performed in MrBayes version 3.2 [46]. Based on the Markov chain Monte Carlo (MCMC) algorithm [47], the best fitting was found to be TVM+F+I substitution model with one million generations, four independent heated chains, and sampling after every 1000 generations [48]. The FigTree ver 1.4.2 was considered for the visualization of the output trees [49].

## Results

### Features of *I*. *monticola* and *I*. *cyanantha*

The genomic libraries generated 4.2–4.9 Gb raw data, which were equivalent to 2.1–2.6 Gb trimmed reads. After sequencing, cutting, and selecting reads, 11,143 and 14,709 contigs were recovered for *I*. *monticola* (Guizhou) and *I*. *monticola* (Yunnan), respectively. Besides, 11,357 and 27,031 contigs were recovered for *I*. *cyanantha* (Guizhou) and *I*. *cyanantha* (Yunnan), respectively. Newly generated complete chloroplast genome sequences were submitted to GenBank under accession numbers MW464331-MW464334. The raw Illumina and PacBio chloroplast sequencing data have been submitted to the NCBI with SRA numbers SUB8890373 and SUB8894092 for *I*. *monticola*, and with SRA numbers SUB8894240 and SUB8894445 for *I*. *cyanantha*. All of these raw data are in the bioprojects PRJNA691973, PRJNA692235, PRJNA692243, PRJNA692246, in the order given.

Contigs mapped to the *I*. *piufanensis* species (GenBank MG162586.1) were then used to reconstruct the *Impatiens*’ chloroplast DNA. The largest plastome was that of *I*. *monticola* (Yunnan) with a length of 152,692 bp, followed by *I*. *monticola* (Guizhou) with 152,656 bp, *I*. *cyanantha* (Guizhou) with 152,391 bp, and *I*. *cyanantha* (Yunnan) with 152,375 bp. Among these Balsaminaceae specimens, the complete lengths ranged from 152,236 bp (*I*. *piufanensis*) to 154,189 bp (*H*. *triflora*) (Table 2 and S1 Table). Newly complete chloroplast genome sequences varied from 152,375–152,692 bp with the long single copy (LSC) region of 83,740, 83,704, 83,284, and 83,275 bp, short single copy (SSC) region of 17,588, 17,532, 17,801, and 17,808 bp and each IR region of 25,664, 25,728, 25,653, and 25,755 bp in *I*. *monticola* (Guizhou), *I*. *monticola* (Yunnan), *I*. *cyanantha* (Guizhou), and *I*. *cyanantha* (Yunnan), respectively.

**Table 2:** Characteristics of complete chloroplast genomes for *Impatiens* specimens.

Specimens	*I*. *monticola (*Guizhou*)*	*I*.*monticola (Yunnan)*	*I*. *cyanantha (Guizhou)*	*I*. *cyanantha (Yunnan)*	*I*.*piufanensis*	*I*.*glandulifera*	*H*. *triflora*
Length (bp)	152,656	152,692	152,391	152,375	152,236	152,260	154,189
LSC (bp)	83,740	83,704	83,284	83,275	83,115	83,261	84,865
IR (bp)	25,664	25,728	25,653	25,755	25,755	25,63	25,622
SSC (bp)	17,588	17,532	17,801	17,808	17,611	17,737	18,080
Total Genes	115	115	115	115	115	113	115
CDS	81	81	81	81	81	80	81
tRNA	30	30	30	30	30	29	30
rRNA	4	4	4	4	4	4	4
Total GC Content (%)	36.7	36.7	36.8	36.8	36.9	36.8	36.9
GC Content in LSC (%)	34.3	34.3	34.5	34.5	34.5	34.5	34.7
GC Content in IR (%)	43.1	43.1	43.1	43.1	43.1	43.1	43.1
GC Content in SSC (%)	29.5	29.5	29.6	29.6	29,3	29,4	29,9

The chloroplast genome length of *I*. *monticola* (Yunnan) was 36 bp longer than that of *I*. *monticola* (Guizhou). Compared with *I*. *monticola* (Yunnan), the chloroplast genome lengths of LSC SSC, and IRs regions of *I*. *monticola* (Guizhou) were longer by 36, 56, and 64 bp, respectively. Besides, the chloroplast genome length of *I*. *cyanantha* (Guizhou) was 16 bp longer than that of *I*. *cyanantha* (Yunnan), while the length of LSC, SSC, and IRs regions of *I*. *cyanantha* (Guizhou) was less than *I*. *cyanantha* (Yunnan) by 9, 7, and 102 bp, in order (Table 1 and S1 Table).

**Fig 1:**
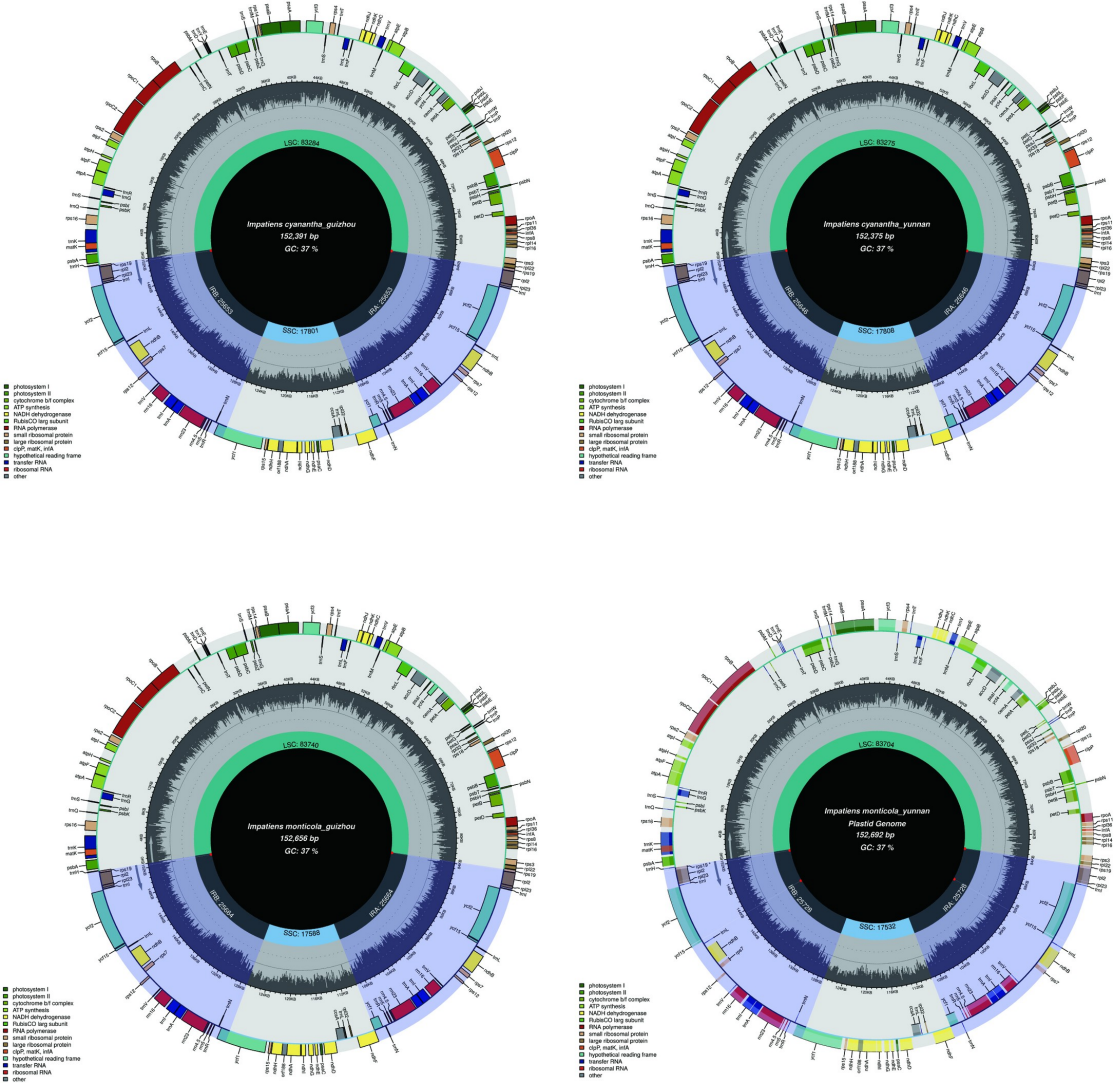
Chloroplast genome structure of *Impatiens* specimens (*I*. *monticola* (Guizhou), *I*. *monticola* (Yunnan), *I*. *cyanantha* (Guizhou), and *I*. *cyanantha* (Yunnan)). The species names and specific information regarding the genome (length, GC content, and the number of genes) were depicted in the center of the plot. In the first inner circle, the optional GC content is depicted as the proportion of the shaded parts of each section. The lengths of the corresponding single short copy (SSC), inverted repeat (IRa and IRb), and large single-copy (LSC) regions were also given. The gradient GC content of the genome is plotted in the second circle with zero levels based on the outer circle.

Besides, the overall guanine-cytosine (GC) contents were very similar in the LSC, SSC, and IRs regions. The GC contents of *I*. *monticola* were an average of 34.3%, 43.1%, and 29.5% in the LSC, IR, and SSC regions, respectively. In the meantime, for *I*. *cyanantha*, the GC contents were average of 34.5%, 43.1%, and 29.6%, respectively (Table 2 and Fig 1).

The physical genetic maps of the newly sequenced *I*. *monticola* (Guizhou), *I*. *monticola* (Yunnan), *I*. *cyanantha* (Guizhou), and *I*. *cyanantha* (Yunnan) are provided in Fig 1 and S1 Fig. The gene content and sequence of these four specimens are almost the same as the previously published data for *I*.*piufanensis*. Like other typical angiosperms, the chloroplast genomes of the *Impatiens* species encoded 115 total distinct genes, including 81 protein-coding, 30 transfer RNA genes (tRNA), and 4 ribosomal RNA genes (rRNA) (Table 3 and S2 Table). As mentioned above, one intron was contained by a total of 8 protein-coding genes (*rps12*, *rpoC1*, *ndhB*, *ndhA*, *rpl*2, *petB*, *atpF*, and *rps16*) and 6 tRNA genes, whereas two introns were contained by two genes (*clpP* and *ycf3*) (Table 3 and S3 Table). The lengths of genes *(rps12*, *ndhA*, *atpF*, rps*16*, *trnK-UUU*, *trnA-UGC*, and *trnI-UAA*) were different in Intron1 of *I*. *cyanantha*, while in *I*. *monticola*, there were only 3 genes (*rps12*, *trnK-UUU*, and *trnA-UGC*) that were different. The lengths of both species were different in the Intron 2 of the *ycf3* gene. Among the introns genes, the *rpoC1* gene had the longest exon (1,626 bp), and *TrnK-UUU* had the longest intron (2,529 bp).

**Table 3:** The list of genes in the chloroplast genomes of *Impatiens* specimens.

Function of Genes	Group of Genes	Gene Names
Photosynthesis-related genes	Rubisco	*rbcL*
	Photosystem I	*psaA psaB psaC psaI psaJ*
	Assembly and stability of Photosystem I	ycf3** *ycf4*
	Photosystem II	*psbA psbB psbC psbD psbE psbF psbH psbI psbJ psbK psbL psbM psbN psbT psbZ*
	ATP synthase	atpA atpB atpE atpF* *atpH atpI*
	Cytochrome b/f complex	petA petB* *petD petG petL petN*
	Cytochrome c synthesis	*ccsA*
	NADPH dehydrogenase	ndhA* ndhB*(2) *ndhC ndhD ndhE ndhFndhG ndhH ndhI ndhJ ndhK*
Transcription and translation-related genes	Transcription	*rpoA rpoB rpoC*1* *rpoC*2
	Ribosomal proteins	rpl2*(2) rpl14 rpl16 rpl20 rpl22 rpl23(2) rpl33 rpl36 rps2 rps3 rps4 rps7(2) rps8 rps11 rps12*(2) rps14 rps15 rps16* *rps18 rps19(2)*
RNA genes	Ribosomal RNA	*rrn4*.*5 rrn5 rrn16 rrn23*
	Transfer RNA	*trnA-UGC*(2) trnC-GCA trnD-GUC trnE-UUC trnF-GAA trnfM-CAU trnG-GCC* trnG-UCC trnH-GUG trnI-CAU*(2) *trnI-GAU*(2) trnK-UUU* *trnL-CAA*(2) trnL-UAG trnL-UAA* *trnM-CAU trnN-GUU*(2) *trnP-UGG trnQ-UUG trnR-ACG*(2) *trnR-UCU trnS-GCU trnS-GGA trnS-UGA trnT-GGU trnT-UGU trnV-GAC*(2) trnV-UAC* *trnW-CCA trnY-GUA*
Other genes	RNA processing	*matK*
	Carbon metabolism	*cemA*
	Fatty acid synthesis	*accD*
	Proteolysis	clpP**
Genes of unknown function	Conserved reading frames	*ycf1 ycf2(2) ycf15(2)*

(2) indicates the m = number of the repeat unit is 2

*Gene contains one intron

**Gene contains two intron.

#### Codon usage

We analyzed the codons in its coding region to determine the genetic information and the relationship between evolution and phylogeny of *Impatiens*. We made a comparison among these seven Balsaminaceae specimens. Codon encoded the genes ranging from 50,745 (*I*. *piufanensis*) to 51,395 (*H*. *triflora*). *I*. *monticola* (Guizhou), *I*. *monticola* (Yunnan), *I*. *cyanantha* (Guizhou), and *I*. *cyanantha* (Yunnan) chloroplast genomes contained 50,885, 50,897, 50,797, and 50,791 codons, respectively (S2 Fig and S4 Table). Besides, in the chloroplast genomes, the leucine was the most frequent amino acid with a percentage of 10.29% and 9.97%, and Cysteines were the least encoded amino acids with only 2.08% and 2.10% in *I*. *monticola* and *I*. *cyanantha*, sequentially.

Relative synonymous codon usage (RSCU) is an excellent indicator for measuring the bias of codon usage in coding sequences. Of the seven Balsaminaceae specimens, *I*. *monticola* had 36 codons of using at equilibrium (RSCU > 1) more frequently than expected. In comparison, the rest of the five Balsaminaceae specimens showed 34 codons in the codon usage bias. Leucine preferred six codon types (CUU, CUG, CUC, UUA, CUA, and UUG). In contrast, the frequency of the start codons (AUG and UGG) encoding methionine and tryptophan exhibited no bias in all Balsaminaceae specimens (S4 Table).

#### Repeat structure analyses

**Fig 2:**
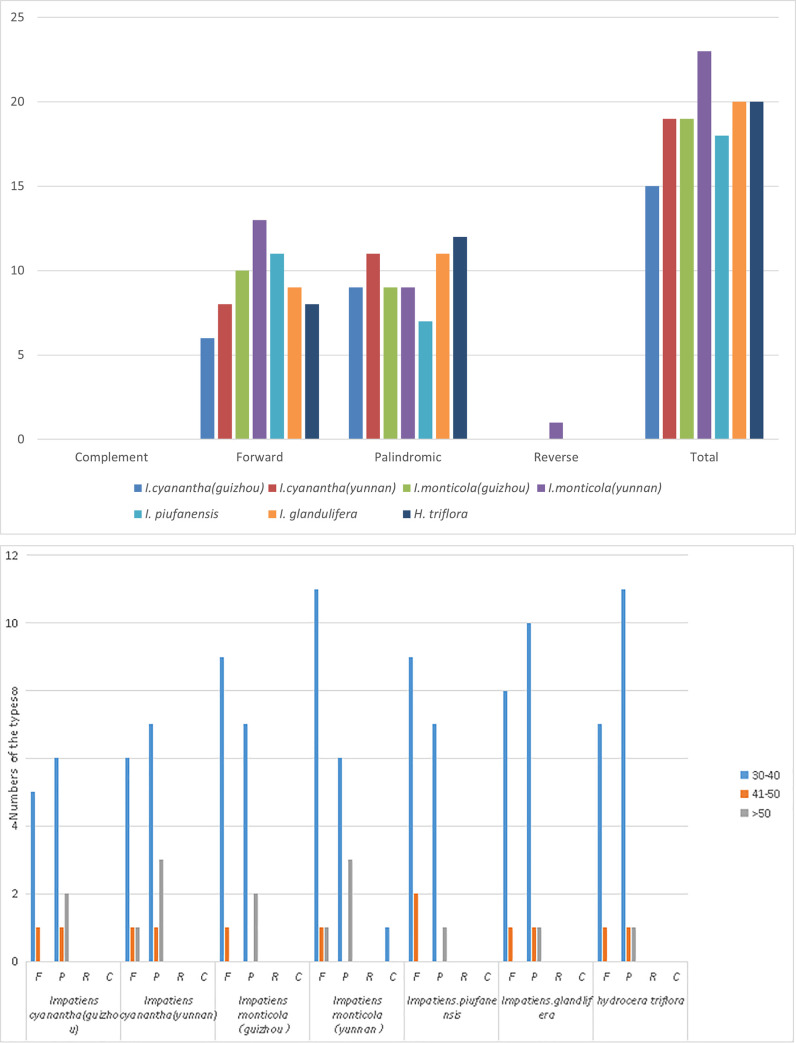
Analysis of repeated sequences in *I*. *monticola* (Guizhou), *I*. *monticola* (Yunnan), *I*. *cyanantha* (Guizhou), *I*. *cyanantha* (Yunnan), *I*. *piufanensis*, *I*. *glandulifera*, and *H*. *triflora*. (a) Total seven species of four repeat types.; (b) Total seven species of four repeat types by length intervals of 30–40; 40–50; >50.

The 141 unique repeats for comparing forward, complement, reverse, and palindromic were examined across Balsaminaceae specimens using REPuter. In detail, there were 19 long repeats (10 forwards, 9 palindromes), 23 long repeats (13 forwards, 9 palindromes, 1 reverse), 15 long repeats (6 forwards, 9 palindromes), 19 long repeats (8 forwards, 11 palindromes) in *I*. *monticola* (Guizhou), *I*. *monticola* (Yunnan), *I*. *cyanantha* (Guizhou), and *I*. *cyanantha* (Yunnan), respectively (Fig 2A and S5 Table). Among all species, palindromic repeats were the most common repeat type. All species contained forward and palindromic repeats; however, no compliment repeats were identified in all Balsaminaceae specimens. A single reverse repeat was only found in *I*. *monticola* (Yunnan). Most of the repeats were less than 40 bp in length; *I*. *monticola* (Yunnan) contained the forwarding repeats in the highest number, while *H*. *trifloral* revealed the palindrome repeats at the highest number of 13 (Fig 2B).

#### Simple sequence repeats analyses

**Fig 3:**
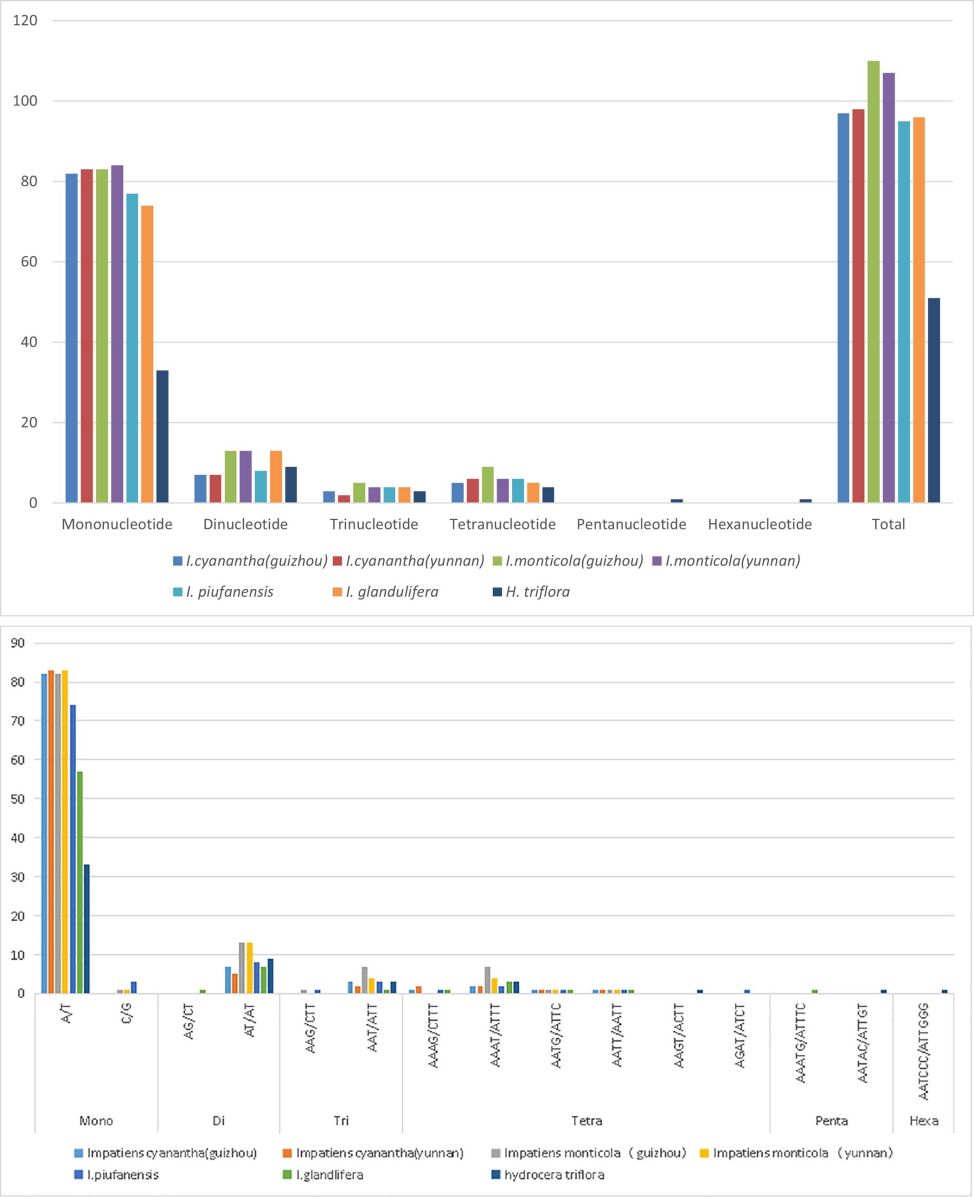
Analysis of simple sequence repeats (SSRs) of *I*. *monticola* (Guizhou), *I*. *monticola* (Yunnan), *I*. *cyanantha* (Guizhou), *I*. *cyanantha* (Yunnan), *I*. *piufanensis*, *I*. *glandulifera*, and *H*. *triflora*. (a) The number of different SSR types; (b) types and frequency of each identified SSR.

The 632 SSRs were performed across the 7 Balsaminaceae plastomes with the MISA online software. The number of SSRs were ranged from 51 (*H*. *triflora*) to 113 [*I*. *monticola* (Guizhou)]. The overall lengths of six types of SSRs were adjusted from 10 to 23 bp (Fig 3A). The most abundant repeats were mononucleotides, which accounted for about 78.89% of the total SSRs. The numbers varied from 33 in *H*. *triflora* to 84 in *I*.*monticola* (Yunnan), followed by Dinucleotide repeats (10.7%), Tetranucleotide repeats (6.2%), Trinucleotide repeats (3.8%). The Penta-nucleotide and Hexanucleotide repeats were the least abundant (1.5%; S6 Table).

Mononucleotide (all A/T), dinucleotide (mostly AT/AT), trinucleotide (AAT/ATT) and tetranucleotide (AAAT/ATTT) SSRs were present in all Balsaminaceae specimens. As mononucleotides (C/G) were only present in *I*.*Monticola* (Guizhou), *I*. *monticola* (Yunnan), and *I*. *piufanensis*, dinucleotides (AG/CT) were only observed in *I*. *glandlifera*. Furthermore, trinucleotide (AAG/CTT) SSRs were only detected in *I*. *monticola* (Guizhou) and *I*. *piufanensis*. On the contrary, hexanucleotides (AATCCC/ATTGGG) were only found in *H*. *trifloral*, and pentanucleotides (AAATG/ATTTC, AATAC/ATTGT) were solely determined in *I*. *glandlifera* and *H*. *trifloral* (Fig 3B). In conclusion, the most abundant type was the mononucleotide SSRs (78.95%) among all 632 SSRs. Additionally, tetranucleotides (AAGT/ACTT) were exclusively seen in *H*. *triflora*, and tetranucleotides (AGAT/ATCT) were unique in *I*. *piufanensis* (S6 Table).

We observed that 20 different SSRs (28.9%) were located in 12 protein-coding genes [*rps16*, *psbK*, *atpF*, *rpoC1*, *rpoB*, *ycf3*, *clpP*(×2), *petB*, *ndhF*, *ccsA*, *ndhD*, *ycf1* (×8)] in *I*. *cyanantha* (Yunnan). For *I*. *cyanantha* (Guizhou), 22 different SSRs (28.9%) were located in 10 protein-coding genes [*rps16*, *atpF*, *rpoC2*(×2), *rpoB*, *clpP*(×2), *rpoA*(×2), *ndhF*, *ndhD*, *ycf1* (×10), and *ndhA*]. Furthermore, SSRs were also detected in CDS regions of the *I*.*monticola* chloroplast genome. It was observed that 21 SSRs (28.9%) were located in 14 genes (CDS) regions [*rps16*(×2), *atpF*, *rpoC2*(×2), *rpoC1*, *psaA*, *ycf3*, *ndhK*, *clpP*(×2), *rpoA*, *ndhF*, *ccsA*, *ndhD*, *ycf1* (×5), and *ndhA*] in *I*. *monticola* (Guizhou). For *I*. *monticola* (Yunnan), 22 different SSRs (28.9%) were located in 14 protein-coding genes [*atpF*, *rpoC2*(×2), *rpoC1*, *rpoB*, *psaA*, *ycf3*, *ndhK*, *clpP*(×2), *rpoA*, *ndhF*, *ccsA*, *ndhD*, *ycf1* (×5), and *ndhA*].

#### Structure in Balsaminaceae chloroplast genomes

**Fig 4:**
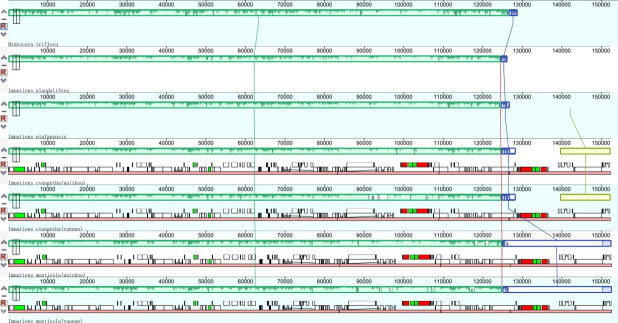
Comparison of sequence arrangement in the chloroplast genomes of the Balsaminaceae specimens.

Most of the angiosperms’ chloroplast genomes are relatively stable; however, the chloroplast genome size and structure may vary based on the different genetic backgrounds and evolutionary histories. The chloroplast genomes were analyzed and compared by the collinear method. The mauve alignment for seven Balsaminaceae specimens revealed optimal collinearity. The collinear blocks of all regions including LSC, SSC, and IRs were relatively conserved, and no gene rearrangement was obtained. Moreover, just as exhibited in the red vertical lines, the structural alignment in Mauve revealed a conserved gene order besides *H*. *triflora*. The yellow vertical lines showed *I*. *cyanantha* (Guizhou) and *I*. *cyanantha* (Yunnan) have the conserved gene order (Fig 4). However, *I*. *monticola* (Guizhou) and *I*. *monticola* (Yunnan) regions, displayed by the blue block, were relatively more conserved.

#### Divergence of protein coding gene sequence

**Fig 5:**
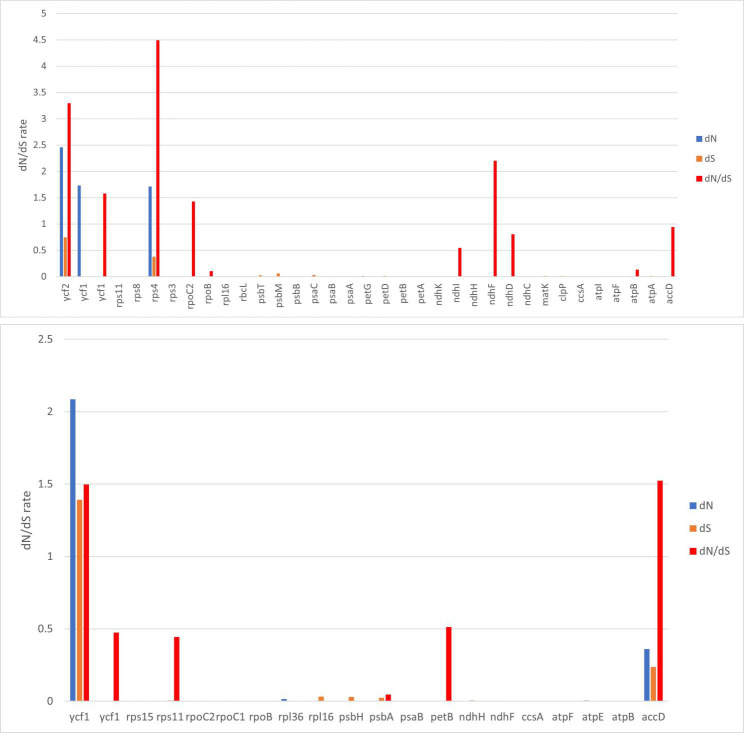
Analysis of dN, dS, and dN/dS rate of *I*. *monticola* and *I*. *cyanantha*. (a) *I*. *cyanantha* (Guizhou) vs. *I*. *cyanantha* (Yunnan); (b) *I*. *monticola* (Guizhou) vs. *I*. *monticola* (Yunnan).

To estimate selection pressures of chloroplast genes, dN, dS, and ω of 80 protein-coding genes were computed and compared in four Balsaminaceae specimens. In a pair of *I*. *cyanantha* (Guizhou) vs. *I*. *cyanantha* (Yunnan), only 10 protein coding genes have ω values with dN values ranging from 0.00049 to 2.45885 and dS values ranging from 0.00065 to 0.74643. When the ω value was within 0.5–1, it contained *accD*, *ndhD*, *ndhI* genes. Meanwhile, the ω value of *rps4*, *ycf2*, *ndhF*, *ycf1*, and *rpoC2* genes just exceeded 1 (Fig 5A). As for *I*. *monticola* (Guizhou) vs. *I*. *monticola* (Yunnan), our comparison showed that only 6 protein-coding genes have ω values with dN values ranging from 0.00049 to 2.0851 and dS values ranging from 0.00187 to 1.39173. Most genes exhibited ω less than 0.5. The *accD* and *ycf1* genes were positively selected to a greater extent than the other genes (Fig 5B). The data suggested that these genes were possibly under positive selection in these endangered. Our analysis identified 5 genes with positive selection sites for *I*. *cyanantha* (Guizhou) vs. *I*. *cyanantha* (Yunnan). These genes included Ribosomal proteins subunit genes (*rps4*), NADPH dehydrogenase subunit genes(*ndhF*), Transcription subunit genes (*rpoC2*), and the *ycf1* and *ycf2* genes. NADPH dehydrogenase is essential during photosynthesis, and transcription subunit genes are encoded and synthesized in the chloroplasts. As for *I*.*monticola* (Guizhou) vs. *I*.*monticola* (Yunnan), *accD* and *ycf1* are positive selection sites.

#### Association analysis between IR expansion and gene loss

**Fig 6:**
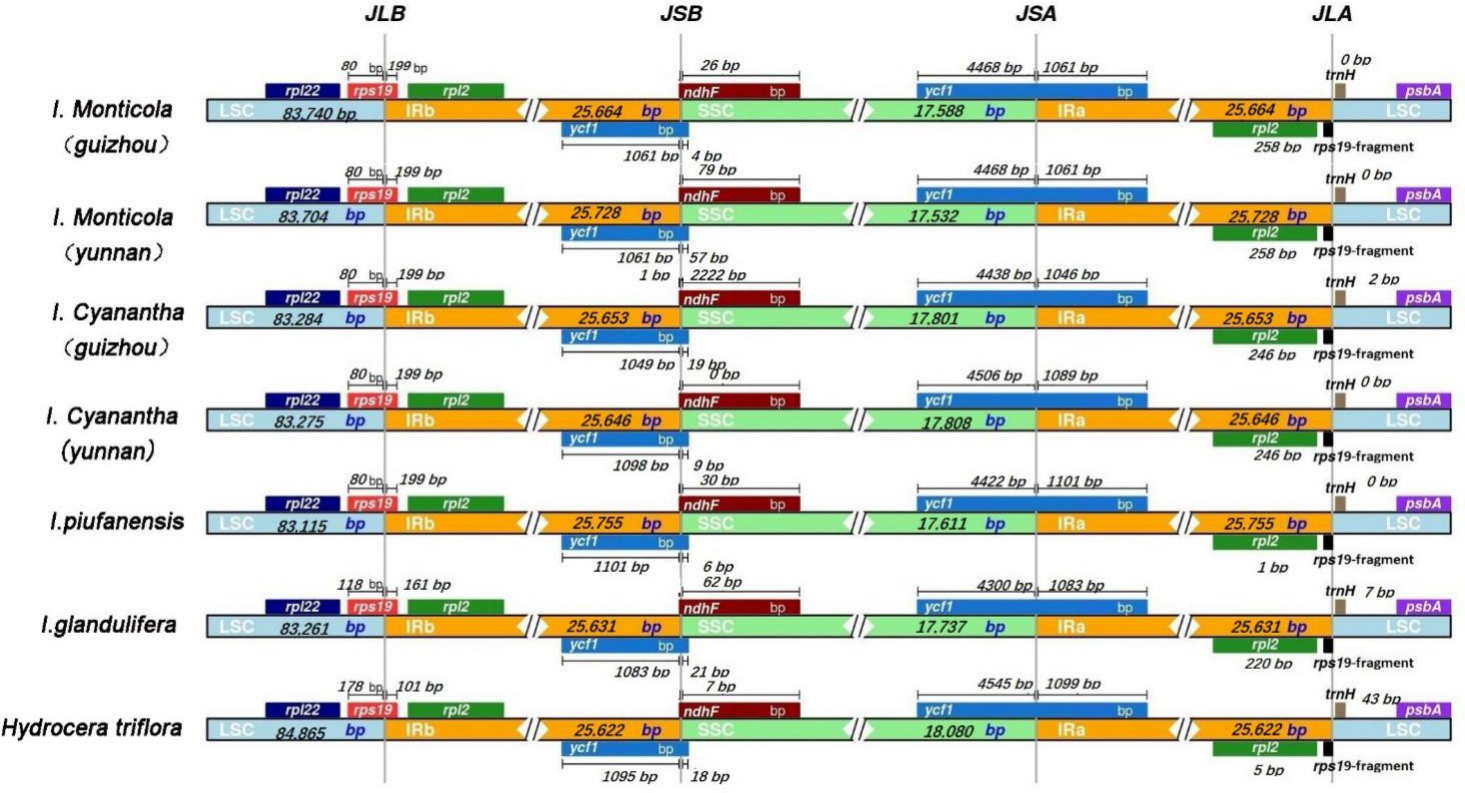
Comparison of the borders of large single copy (LSC), small single copy (SSC), an inverted repeat (IR) regions.

The four junctions in the regions are presented in Fig 6 by a detailed structure comparison among the Balsaminaceae specimens. The IRB-LSC junction (JLB) is located in the *rps19* gene, which intervened between the IRB and LSC region. However, among *I*. *monticola* (Guizhou), *I*. *monticola* (Yunnan), *I*. *cyanantha* (Guizhou), *I*. *cyanantha* (Yunnan), and *I*. *piufanensis*, the lengths of *rps19* in the IRB and LSC region were the same, while the *rps19* lengths of *I*. *glandulifera* and *H*. *triflora* in the LSC region were 118 bp and 178 bp, respectively. The SSC-IRB junction (JSB) was located or adjacent to *ycf1* and *ndhF*; seven specimens were all located just adjoined at the end of *ycf*1 from 1049bp to 1101bp. The overlap between *ndhF* and *ycf1* was detected in *I*. *cyanantha* (Guizhou), in which *ndhF* expanded into the IRB region for 1 bp. Besides, for *I*. *monticola* (Guizhou), *I*. *monticola* (Yunnan), *I*. *cyanantha* (Yunnan), *I*. *piufanensis*, *I*. *glandlifera* and *H*. *triflora*, the distances between *ndhF* and JSB were 26 bp, 79 bp, 0 bp, 30 bp, 62 bp, and 7 bp, in the order given. The IRA-SSC junction (JSA) is located in the *ycf1*, which covered the IRA and SSC region. The length of *ycf1* in the SSC region varied from 4,300 bp to 4,545 bp. The LSC-IRA junctions (JLA) were located between *rpl2* and *traH*. The distances between *traH* and JLA of *I*. *cyanantha* (Guizhou), *I*. *glandlifera*, and *H*. *triflora* were 2 bp, 7 bp, and 43 bp, respectively while the lengths of *I*. *monticola* (Guizhou), *I*. *monticola* (Yunnan), *I*. *cyanantha* (Yunnan), *I*. *piufanensis* in the LSC region were all 0 bp.

**Fig 7:**
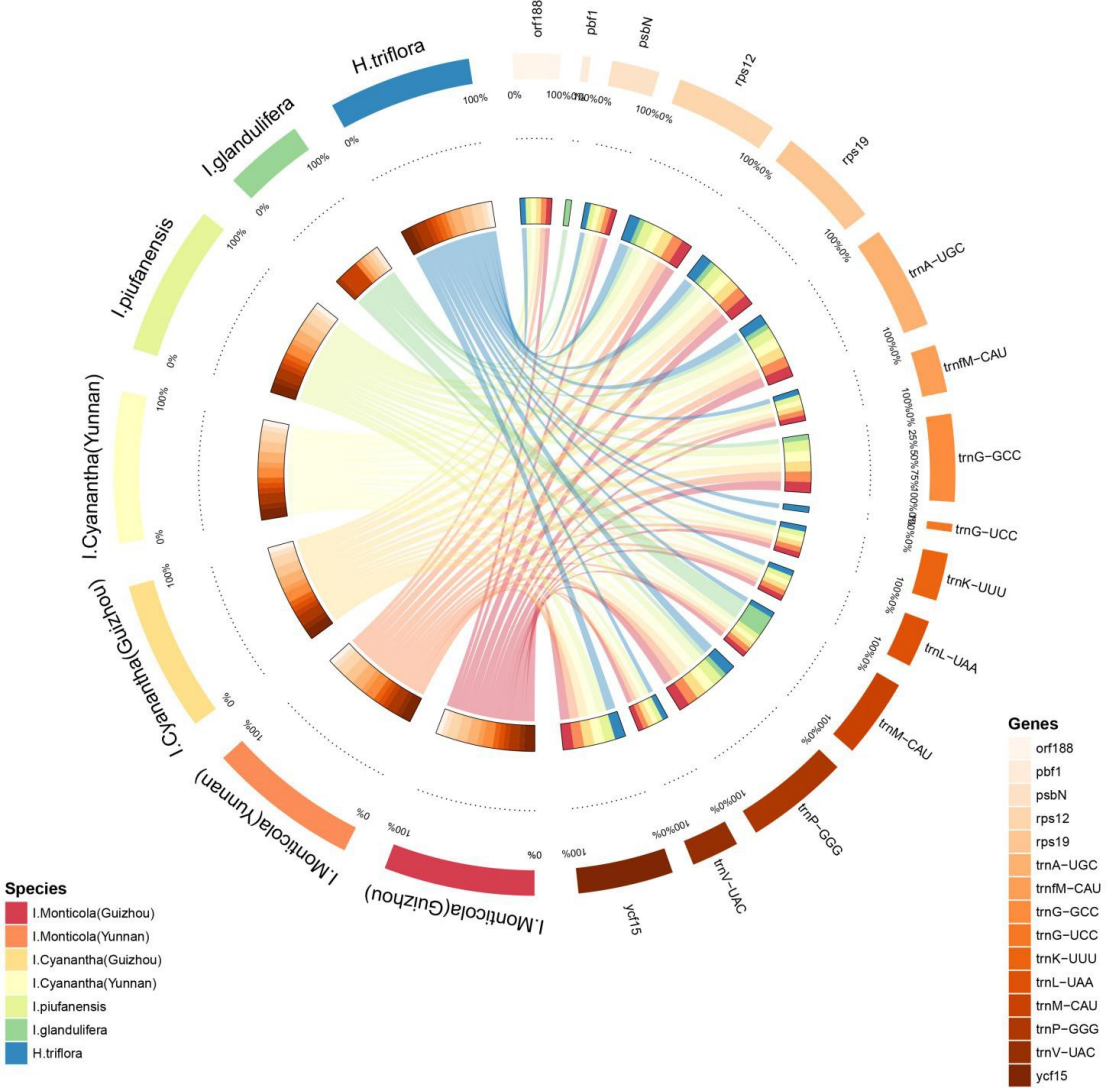
Alignment of the gene loss in the seven chloroplast genomes by using Circos plot.

The pseudogenization of *orf188*, as well as the deletion of *psbN*, *trnfM-CAU*, *trnG-GCC*, *trnK-UUU*, *trnL-UAA*, *trnP-GGG*, *trnV-UAC*, *and ycf15*, were observed in *I*. *glandulifera* (Fig 7). The additional loss of *trnG-GCC*, as well as the pseudogenization of *trnG-UCC*, were only identified in *H*. *triflora*. Also, the loss of *trnP-GGG* was observed in *I*. *piufanensis*. As the result of gene loss and pseudogenization, a total of 108,112 putatively functional genes were observed in *I*. *glandulifera* and *H*. *triflora*. The pseudogenization *pbf1* was solely detected in *I*. *glandulifera*.

#### Comparative genomic divergence and genome rearrangement

**Fig 8:**
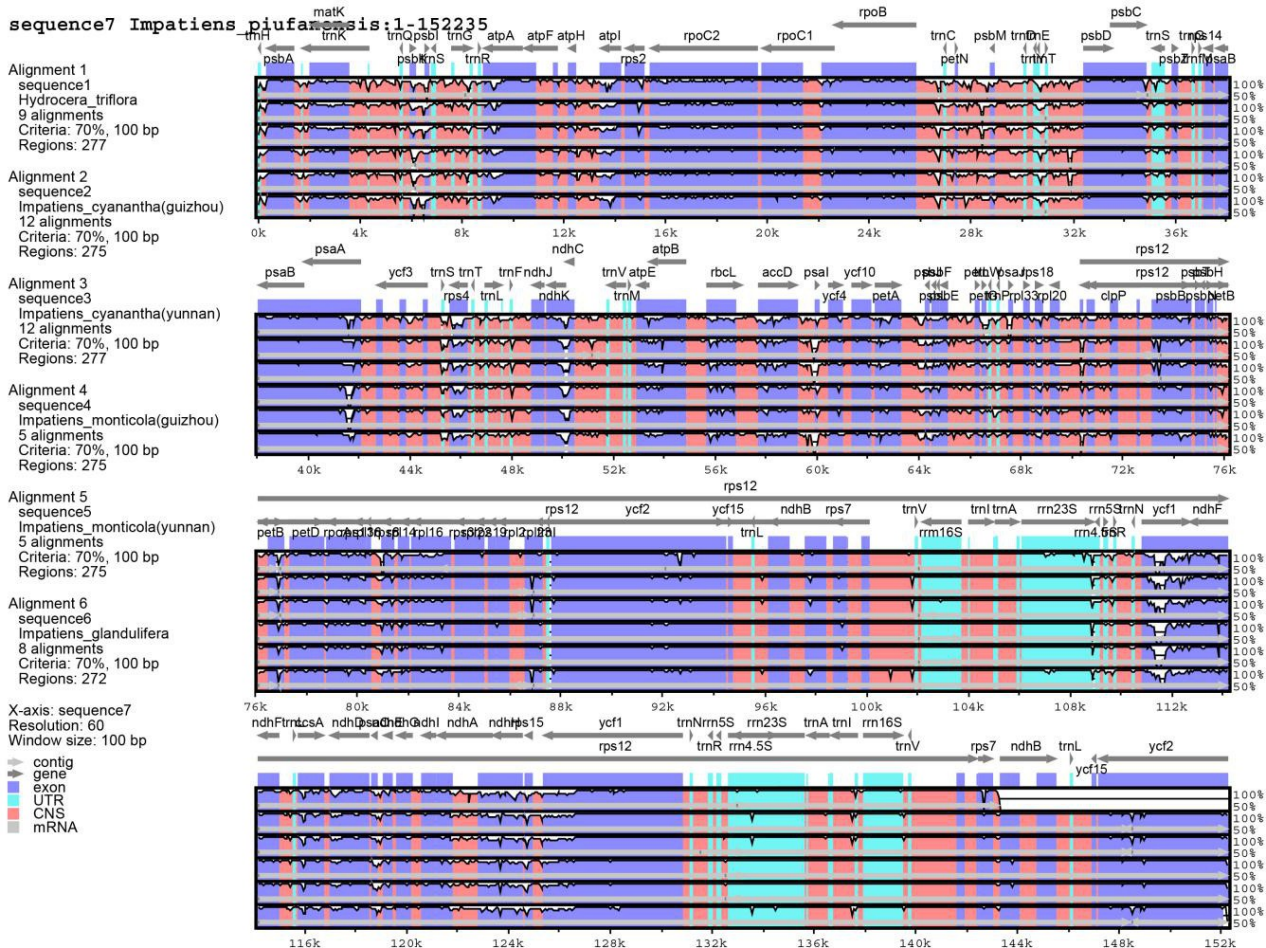
Alignment of the seven chloroplast genomes with *I*. *piufanensis* as a reference by using mVISTA.

Using the mVISTA software, the hyper-variable regions were detected and compared with the whole chloroplast genomes. The *I*. *piufanensis* was selected as the reference genome. However, *H*. *triflora* and other *Impatiens* species showed sequence divergences such as *matK*, *psbK*, *atpH-atpI*, *trnC-trnT*, *petN*, *psbM*, *atpE*, *rbcL*, *accD*, *psaL*, *rps3-rps19*, *rpoB*, *rpl16*, *ndhB*, *ndhF*, *ndhG-ndhA*, *ycf1*, *a*nd *ndhH* (Fig 8). In the SSC region, the three most divergent genes were *ndhF*, *ycf1*, *and ndhH*. The *psbK-psbI*, *atpI*, and *rps4-trnF* genes showed some sequence divergence in *I*. *piufanensis*, *I*. *glandlifera*, and *H*. *triflora*.

**Fig 9:**
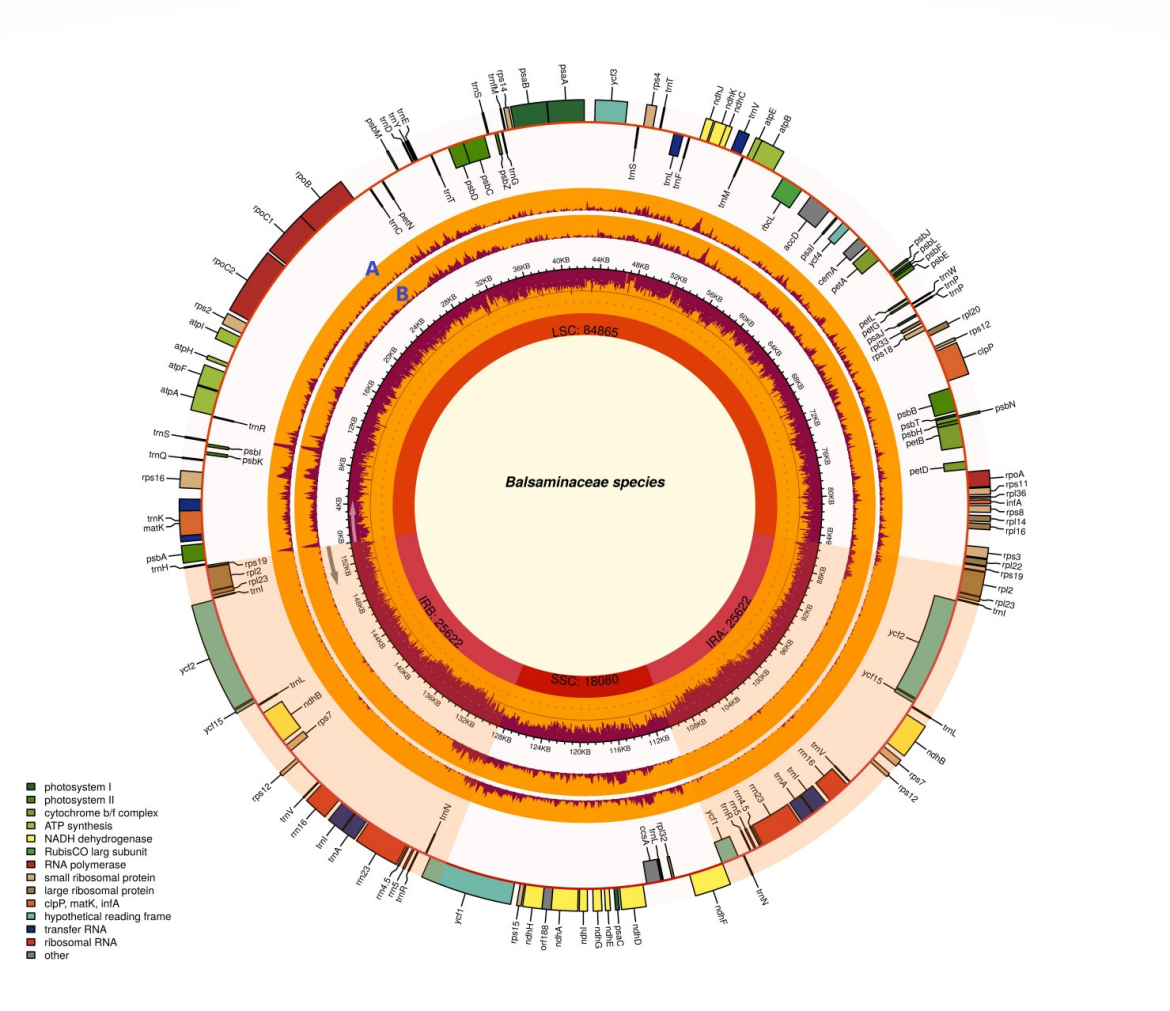
Chloroplast genome map of Balsaminaceae specimens. Extending outward, the next two layers are the optional input indices of the nucleotide of *Impatiens* species compared with *H*. *triflora*. (A) based on the chloroplast genomes of Balsaminaceae specimens with *H*. *triflora*; (B) based on the chloroplast genomes of Balsaminaceae specimens without *H*. *triflora*.

Furthermore, software DnaSP was used to detect the highly variable regions by sliding window analysis of all the Balsaminaceae specimens, including *H*. *trifloral* (Fig 9 and S7 Table). The nucleotide variability (Pi) ranged from 0. 01% (*rrn23*) to 9.0% (*ycf1*). In addition, *ycf1*, *trnT-UGU*, *trnS-GCU*, *rps16*, *rpl32*, *rps15*, *rpl33*, *ndhC*, *trnC-GCA*, *psbM-trnD-GUC*, *trnG-GCC* and *petA-psbJ* showed remarkably high values (Pi > 0.06) (Fig 9). The *ycf1* gene demonstrated the highest average sequence divergence (0.090), followed by *trnT-UGU* (0.078) and *trnS-GCU* (0.074; Fig 9). Similarly, we detected the sequence divergence without *H*. *triflora*. Thereby, only five regions (*ycf1*, *trnQ-UUG*, *rpl32*, *rps16*, and *trnS-GCU*) had tremendously high values.

#### Phylogenetic analysis

**Fig 10:**
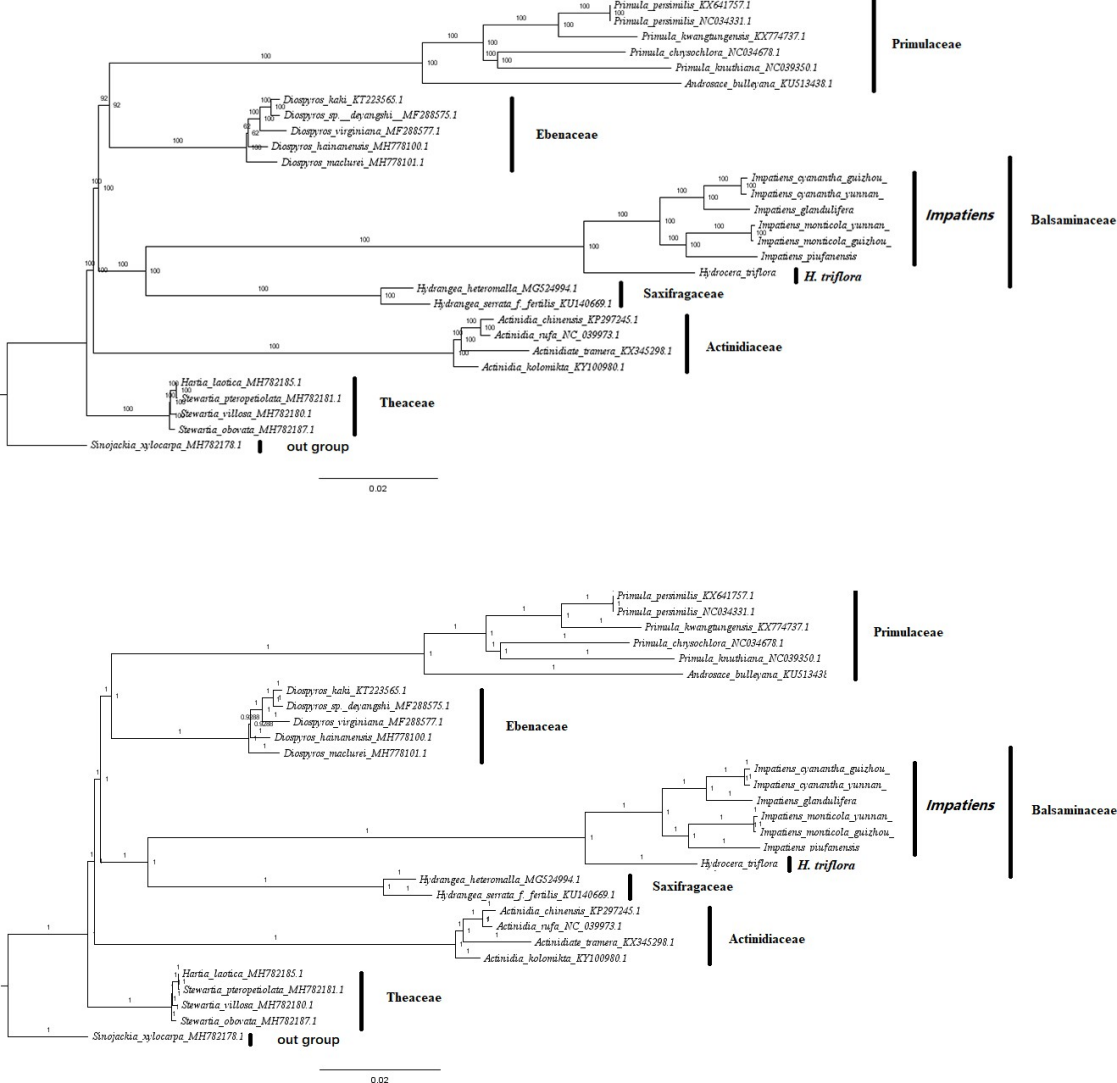
Phylogenetic tree of *Impatiens* species within the Balsaminaceae. The entire genome data set was analyzed using maximum likelihood and Bayesian information. The numbers above and below the branches represent bootstrap values in the ML and BI trees. The green color represents the positions of Impatiens species. (a) ML tree; (b) BI tree.

Based on the complete chloroplast genomes, we used the phylogenetic tree to explore the phylogenetic positions and evolutionary relationships of *I*. *cyanantha* and *I*. *monticola* species (S8 Table). These chloroplast genomes from seven families: seven Balsaminaceae, six Primulaceae, five Ebenaceae, four Theaceae, two Saxifragaceae, four Actinidiaceae, and one Styracaceae specimen(s) as outgroups. The two datasets (ML and BI) topologies generated a similar structure (Fig 10). The three selected families (Actinidiaceae, Theaceae, and Styracaceae) were clustered into a monophyletic branch. The Genus Primula and Androsace of the family Primulaceae were clustered into a clade, while the family Theaceae consisted of the Stewartia and the Hartia Dunn. But the Balsaminaceae and Saxifragaceae were clustered into a monophyletic branch. All Balsaminaceae specimens formed a monophyletic subclade in both trees. The support values in the ML tree were 100% in both datasets; moreover, both showed a sister relationship with *I*. *monticola* (Guizhou) and *I*. *monticola* (Yunnan), formed a clade with *I*. *piufanensis* indicating their close connection. In the meantime, *I*. *cyanantha* (Guizhou) and *I*. *cyanantha* (Yunnan) formed a clade with *I*. *glandlifera* (Fig 10).

## Discussion

In this study, we assembled the chloroplast genome of two phenotypic species (*I*. *cyanantha* and *I*. *monticola*), which are found at different altitudes in Guizhou and Yunnan, China. The basic features of the gene were highly conserved, but the expansion and contraction of the IR region have resulted in minor changes in the boundary and size of the chloroplast genome, thus increasing the chloroplast genetic diversity and evolutionary events (Table 1 and S1 Table). Moreover, a comparative analysis of *I*. *cyanantha* from Guizhou and Yunnan revealed minor changes in length. While the IR region of *I*. *cyanantha* (Guizhou) had lost entirely 64 bp in the *ycf1*, the LSC and SSC regions in *I*. *cyanantha* (Guizhou) were 36 bp and 56 bp more than *I*. *cyanantha* (Yunnan), in the order given. And the SSC region of *I*. *monticola* (Guizhou) had completely lost 7 bp in the *ycf1* pseudogene, while the LSC and IR regions in *I*. *monticola* (Guizhou) were 9 bp and 7 bp more than *I*. *monticola* (Yunnan), respectively. The *ycf1* pseudogene may be due to the length of the chloroplast genome between *I*. *monticola* (Guizhou) and *I*. *monticola* (Yunnan). In *I*. *cyanantha* (Guizhou) and *I*. *cyanantha* (Yunnan), the *ycf1* pseudogene in the IRB region and the *ycf1* gene in the IRA region may cause a variation in length [50].

Among the 5 Balsaminaceae specimens, the chloroplast genome ranged from 152,236 bp (*I*. *piufanensis*) to 154,189 bp (*H*. *triflora*), the overall GC content ranged from 36.70% (*I*. *monticola*) to 36.90% (*H*. *triflora*) and contained 114 distinct genes including 81 PCGs, 29 tRNA, and 4 rRNA genes; also, one or two introns were found among these 16 genes except for *I*. *glandulifera*. The results for *I*. *cyanantha* and *I*. *monticola’s* chloroplast genome were consistent with the previous analysis. Like other angiosperms, a high GC content was often associated with the degree of the primitiveness of a taxon. Furthermore, conforming with that observed *I*. *cyanantha* and *I*. *monticola*, introns play a vital role in selective gene-splicing. The *trnK-UUU* had the largest intron, while the *trnL-UAA* had the smallest intron. The gain of the intron was usually considered to have a close relationship with the evolution of photosynthesis. However, no introns were lost in the Balsaminaceae specimens, which indicated that the chloroplast genome was highly conserved during evolution and development.

Simple sequence repeats (SSRs), as one of the primary sources of molecular markers, have been recognized for having a high polymorphism rate and abundant variation [51]. This study detected that 95–110 SSRs were distributed in the *Impatiens* species (Fig 3 and S6 Table). Moreover, pentanucleotide and hexanucleotide repeats were only identified in *H*. *triflora*. Meanwhile, tandem G or C repeats were rarely contained here in the cpSSRs except for *I*.*monticola*. Similarly, among these chloroplast genomes, most divergent genes were detected [52], especially the SSRs of *I*.*cyanantha* showed abundant variation. We observed that *psbK*, *ycf3*, *petB*, and *ccsA* were only located in *I*.*cyanantha* (Yunnan) while the *rpoC2*, *rpoA*, and *ndhA* were only found in *I*.*cyanantha* (Guizhou). The SSRs of *I*.*monticola* also presented abundant variation, *rps16* was only located in *I*.*monticola* (Guizhou) while the *rpoB* was solely detected in *I*.*monticola* (Yunnan). This strong evidence indicates that abundant variation of SSRs loci is useful for species identification at the population, intraspecific, and cultivar levels and phylogenic study (Fig 7). The IRs regions in all Balsaminaceae chloroplast genomes showed less divergence than the SSC and LSC regions.

Moreover, our analysis identified 16 genes with different SSRs. These genes were named as ribosomal proteins (*rps16*), photosystem II subunit (*psbK*), photosystem I subunit (*psaA*), ATP synthase subunit (*atpF*), cytochrome b/f complex subunit (*petB*), cytochrome c synthesis subunit (*ccsA*), assembly and stability of photosystem I subunit (*ycf3*), three NADPH dehydrogenase subunit (*ndhD*, *ndhA*, and *ndhF*), four transcription subunit (*rpoA*, *rpoC1*, *rpoB*, *rpoC2*), *clpP* and *ycf1* genes. In general, photosynthesis-related genes are essential for plant cells, and the primary function of its product is the degradation of polypeptides. We identified different SSRs in the photosynthesis-related genes in our study, which might have played critical roles in the adaptive evolution of *Impatiens* species. A previous study showed that plants had various adaptive strategies under unpredictable environmental conditions. For instance, the extreme environments, particularly the cold temperatures and high irradiation, were unsuitable for the efficient photosynthesis of plants. Therefore, a set of photosynthetic protection strategies were desired for survival and reproduction in high altitude plants. Therefore, in general, adaptive evolution is evident and clear. *Impatiens* species were mainly distributed in the Yunnan-Guizhou Plateau and adjacent regions. Hence, these different regions’ species might also have some mechanisms to adapt to extreme environments.

Synonymous and nonsynonymous nucleotide substitution patterns are significant markers for gene evolution studies. In most genes, synonymous nucleotide substitutions have occurred more frequently than the nonsynonymous ones. A ratio of dN/dS < 1 indicates purifying selection, dN/dS > 1 denotes probable positive selection, and dN/dS values close to one indicate neutral evolution. In this study, 5 genes with positive selection sites were identified in *rps4* (4.49), *ycf2* (3.29), *ndhF* (2.20), *ycf1* (1.58) and *rpoC2* (1.42) in *I*. *cyanantha* (Guizhou) vs. *I*. *cyanantha* (Yunnan), and *accD* (1.52) and *ycf1* (1.49) in *I*. *monticola* (Guizhou). vs. *I*. *monticola* (Yunnan). These genes included ribosomal proteins subunit (*rps4*), NADPH dehydrogenase subunit (*ndhF*), Transcription subunit (*rpoC2*), *ycf1*, *accD*, and *ycf2* genes. A previous study showed that dN/dS played a significant role in understanding the dynamics of molecular evolution of plant species.

Similarly, *rps4* and *ndhF*, encoding ribosomal protein subunits and NADPH dehydrogenase subunits, played an important role in the life history of the plant. Besides, the *rpoC2* gene are encoding Transcription subunits. For instance, *ycf1* and *accD* genes had been proven to the fast evolution gene. The *accD* gene could affect plant fitness and leaf longevity and the *ycf1* gene had been classified as the most divergent one in the plastomes. A previous study showed that plants had various adaptive strategies under unpredictable environmental conditions. For instance, extreme environments, particularly the cold temperatures and high irradiation, may have some protection strategies for survival and reproduction in high altitude plants. Consequently, these results indicated that these genes might be under positive selection to adapt to the specific ecological environment during the evolution.

In the present study, based on the Bayesian information (BI) and the maximum likelihood (ML) trees, the results showed the same. The seven families could be classified into five monophyletic clades (Fig 10). Actinidiaceae was the basal group in the phylogenetic trees. The Primulaceae and Ebenaceae were gathered into one clade, and the Balsaminaceae was sister to Saxifragaceae. Most species from the same genus were clustered together. Besides, all Balsaminaceae specimens formed a monophyletic subclade in both trees. *H*. *triflora* and *Impatiens* species formed two subclades (Fig 10). *H*. *triflora* was located at the bottom of the phylogenetic trees and all *Impatiens* species were clustered into another clade. *I*. *monticola* and *I*. *piufanensis* species with the most similar morphological characteristics were clustered together, suggesting that the two species were very likely to be experienced in the same habitat and evolutionary process. The chloroplast genome of two phenotypically species, *I*. *cyanantha* and *I*. *monticola*, which were from different altitudes and regions, were clustered into a monophyletic branch. Thus, the resulting phylogenomic tree highly supported that the Balsaminaceae specimens formed a monophyletic subclade, which is consistent with the results of plastid genes and supports the classification of Ericicales in the updated APG IV system. Furthermore, the results indicate that using the whole chloroplast genome sequence is feasible to analyze the systematic evolution.

These results indicate that the whole chloroplast genome, LSC, SSC, and IRs regions vary slightly in different altitudes and regions. As we know, altitude provides substantial changes in temperature, atmospheric pressure, UV-B radiation, and humidity [53]. Consequently, in response to such climatic variations, plants have to regulate their physiological processes and modify their phenotypic traits based on different environmental changes across altitudes [54]. Therefore, the length of the chloroplast genome is seen as one of many adaptations of plants to climate conditions [55]. The altitude and topography environment can determine the plant performance and leaf morphological traits [56]. Besides, plant ecotypes and phenotypic plasticity were associated with variation of altitudinal patterns in leaf traits [57].

## Conclusions

In the present study, the complete chloroplast genomes of *I*. *monticola* and *I*. *cyanantha* were analyzed. We compared *I*. *monticola* and *I*. *cyanantha* with the other three selected Balsaminaceae specimens. The gene size, content, and order had minor differences. The contraction and expansion of the IR boundary regions showed the chloroplast genome size variation. Additionally, the highly variable regions were in *ycf1*, *trnT-UGU*, *trnS-GCU*, *rps16*, *rpl32*, *rps15*, *rpl33*, *ndhC*, *trnC-GCA*, *psbM-trnD-GUC*, *trnG-GCC*, and *petA-psbJ*, which can provide genetic information for the creation of potential molecular markers and genetic diversity. Meanwhile, in a pair of *I*. *cyanantha*, the *rps4*, *ycf2*, *ndhF*, *ycf1*, and *rpoC2* genes underwent positive selection. As for *I*. *monticola*, the *accD* and *ycf1* genes were positively selected. The phylogenetic analysis produced the trees with similar topology. *I*. *monticola* and *I*. *piufanensi* formed a clade with a more comparable relationship than *I*. *cyanantha* with *I*. *glandlifera*. Generally, this study might contribute to further research on Impatiens species’ phylogeny, taxonomy, genetic engineering studies and provide some possible significant information about Impatiens’ systematics and evolution.

## Supporting information

S1 Table Complete chloroplast genomes for 7 Balsaminaceae specimens. (XLSX)

S2 Table Distribution of genes and intergenic regions for 7 specimens in Balsaminaceae. (XLSX)

S3 Table The genes having intron in the 7 Balsaminaceae specimens chloroplast genomes. (XLSX)

S4 Table Codon content of amino acid and stop codon of 7 Balsaminaceae specimens. (XLSX)

S5 Table The comparison of long repeats among 7 Balsaminaceae specimens. (XLSX)

S6 Table The comparison of SSRs among 7 Balsaminaceae specimens. (XLSX)

S7 Table The Pi value of 7 Balsaminaceae specimens. (XLSX)

S8 Table The GenBank accession numbers of 29 specimens using in phylogenetic analysis. (DOCX)

S1 Fig Chloroplast genome structure of two phenotypically species: *I*. *cyanantha* and *I*. *monticola* from Guizhou and Yunnan. (DOCX)

S2 Fig Codon content of 20 amino acid and stop codons in all protein-coding genes of the chloroplast genomes of seven Balsaminaceae specimens. (DOCX)
