# Supplementary material for: Complete chloroplast genomes of Impatiens cyanantha and Impatiens monticola: Insights into genome structures, mutational hotspots, comparative and phylogenetic analysis with its congeneric species
Source: PLoS One. 2021 Apr 2;16(4):e0248182. doi: 10.1371/journal.pone.0248182 (PMC8018631; doi:10.1371/journal.pone.0248182)
Supplement: S8 Table — (DOCX) [file pone.0248182.s008.docx]

| **S8 Table. The GenBank accession numbers of 29 species using in phylogenetic analysis** | | | |
| --- | --- | --- | --- |
| Number | Species | | Genbank number |
| 1 | *Stewartia* | *villosa* | MH782180.1 |
| 2 | *Stewartia* | *pteropetiolata* | MH782181.1 |
| 3 | *Hartia* | *laotica* | MH782185.1 |
| 4 | *Stewartia* | *obovata* | MH782187.1 |
| 5 | *Sinojackia* | *xylocarpa* | MH782178.1 |
| 6 | *Diospyros* | *virginiana* | MF288577.1 |
| 7 | *Diospyros* | *deyangshi* | MF288575.1 |
| 8 | *Diospyros* | *kaki* | KT223565.1 |
| 9 | *Diospyros* | *maclurei* | MH778101.1 |
| 10 | *Diospyros* | *hainanensis* | MH778100.1 |
| 11 | *Hydrangea* | *serrata fertilis* | KU140669.1 |
| 12 | *Hydrangea* | *heteromalla* | MG524994.1 |
| 13 | *Primula* | *persimilis* | NC034331.1 |
| 14 | *Primula* | *persimilis* | KX641757.1 |
| 15 | *Primula* | *kwangtungensis* | KX774737.1 |
| 16 | *Primula* | *chrysochlora* | NC034678.1 |
| 17 | *Primula* | *knuthiana* | NC039350.1 |
| 18 | *Androsace* | *bulleyana* | KU513438.1 |
| 19 | *Impatiens* | *Monticola（Guizhou)* | MW464333 |
| 20 | *Impatiens* | *Monticola（Yunnan)* | MW464334 |
| 21 | *Impatiens* | *Cyanantha(Guizhou)* | MW464331 |
| 22 | *Impatiens* | *Cyanantha(Yunnan)* | MW464332 |
| 23 | *Impatiens* | *glandulifera* | MK358447.1 |
| 24 | *Hydrocera* | *triflora* | MG162585.1 |
| 25 | *Actinidiate* | *tramera* | KX345298.1 |
| 26 | *Actinidia* | *rufa* | NC39973.1 |
| 27 | *Actinidia* | *chinensis* | KP297245.1 |
| 28 | *Actinidia* | *kolomikta* | KY100980.1 |
| 29 | *Impatiens* | *piufanensis* | MG162586.1 |
