# Supplementary material for: Complete chloroplast genomes of Impatiens cyanantha and Impatiens monticola: Insights into genome structures, mutational hotspots, comparative and phylogenetic analysis with its congeneric species
Source: PLoS One. 2021 Apr 2;16(4):e0248182. doi: 10.1371/journal.pone.0248182 (PMC8018631; doi:10.1371/journal.pone.0248182)
Supplement: S1 Fig — (DOCX) [file pone.0248182.s009.docx]

| **S1 Fig. Chloroplast genome structure of two phenotypically species, *I. cyanantha* from Guizhou** |
| --- |


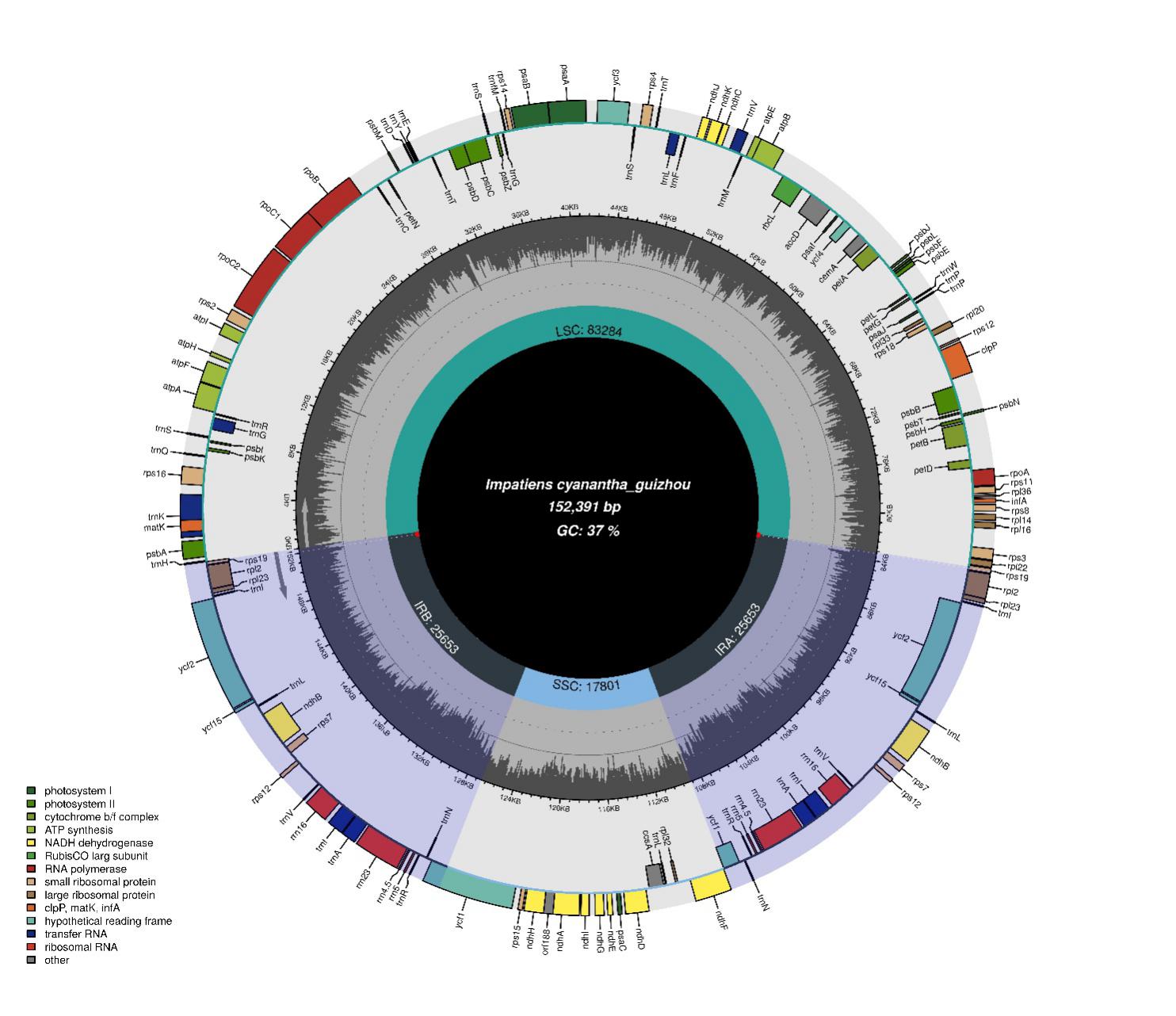


**Fig 1. Chloroplast genome structure of *Impatiens specimens I. cyanantha* from Guizhou*.*** The species name and specific information regarding the genome (length, GC content, and the number of genes) are depicted in the center of the plot. In the first inner circle, the optional GC content is depicted as the proportion of the shaded parts of each section. The length of the corresponding single short copy (SSC), inverted repeat (IRa and IRb), and large single-copy (LSC) regions is also given. The gradient GC content of the genome is plotted in the second circle with zero level based on the outer circle.

**Figure S1 Chloroplast genome structure of two phenotypically species, *I. cyanantha* from Yunnan**


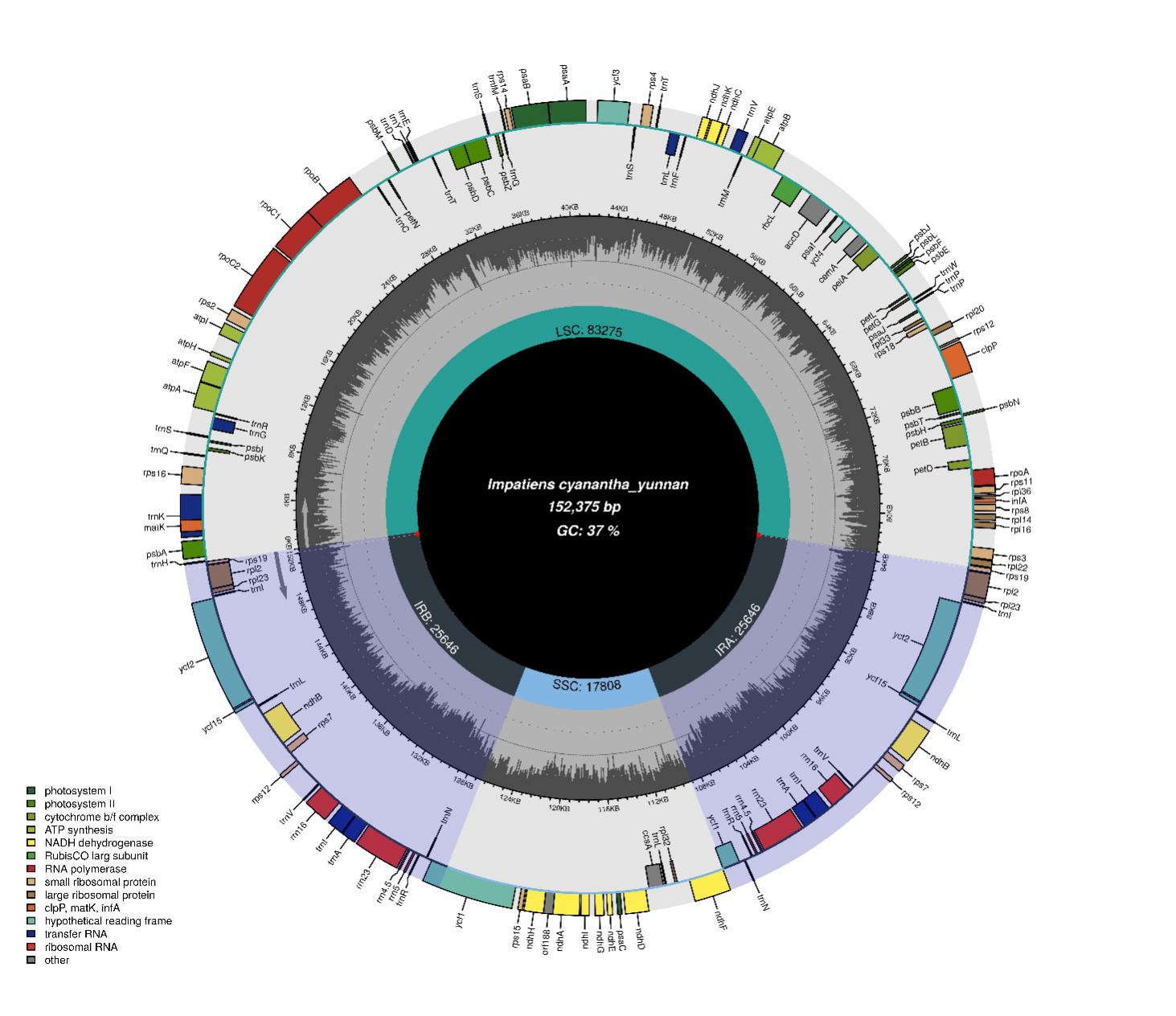


**Fig 1. Chloroplast genome structure of *Impatiens* specimens *I. cyanantha* from Yunnan*.*** The species name and specific information regarding the genome (length, GC content, and the number of genes) are depicted in the center of the plot. In the first inner circle, the optional GC content is depicted as the proportion of the shaded parts of each section. The length of the corresponding single short copy (SSC), inverted repeat (IRa and IRb), and large single-copy (LSC) regions is also given. The gradient GC content of the genome is plotted in the second circle with zero level based on the outer circle.

**Figure S1 Chloroplast genome structure of two phenotypically species, *I. monticola* from Yunnan**


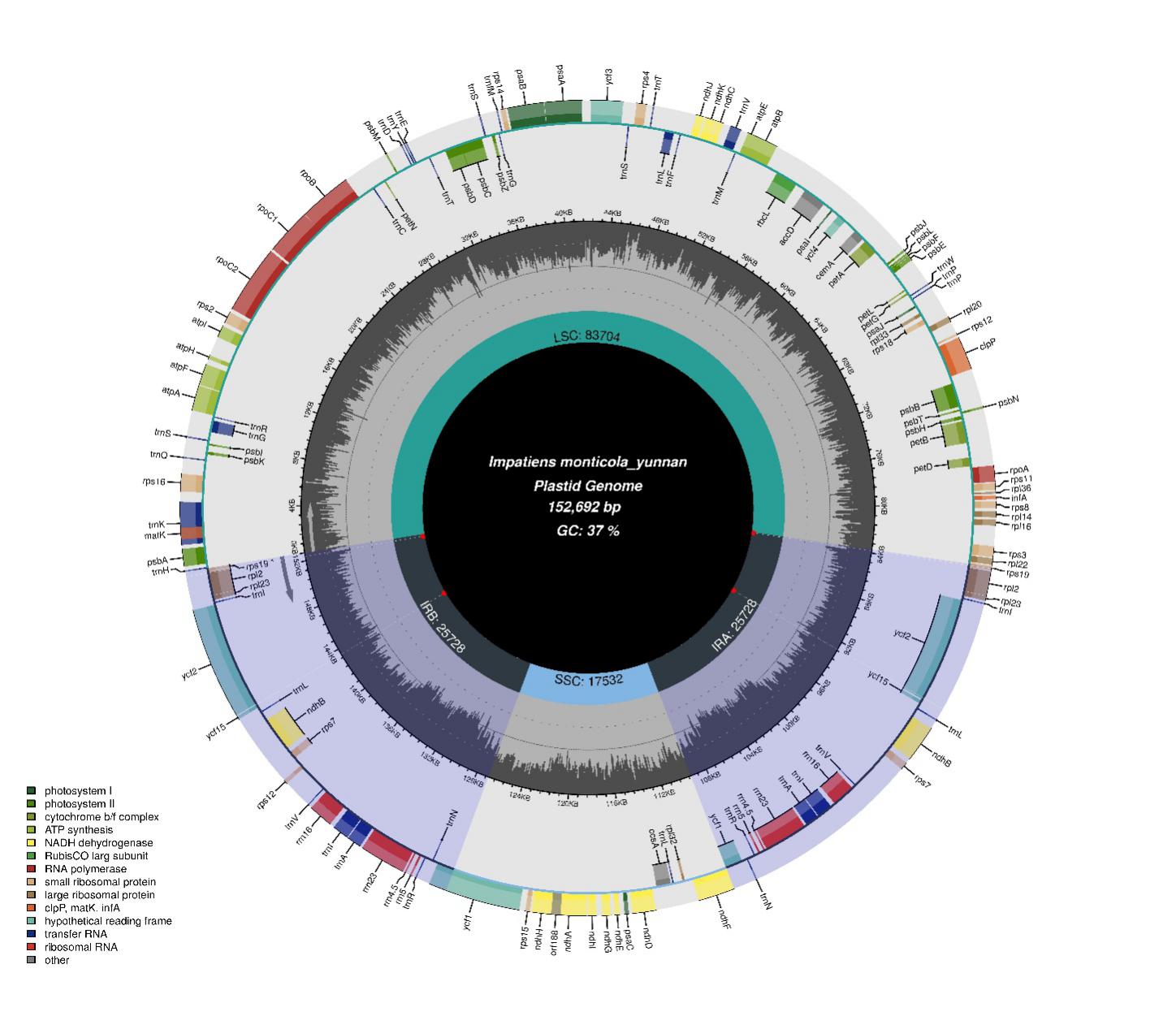


**Fig 1. Chloroplast genome structure of *Impatiens* specimens *I. monticola* from Yunnan*.*** The species name and specific information regarding the genome (length, GC content, and the number of genes) are depicted in the center of the plot. In the first inner circle, the optional GC content is depicted as the proportion of the shaded parts of each section. The length of the corresponding single short copy (SSC), inverted repeat (IRa and IRb), and large single-copy (LSC) regions is also given. The gradient GC content of the genome is plotted in the second circle with zero level based on the outer circle.

**Figure S1 Chloroplast genome structure of two phenotypically species, *I. monticola* from Guizhou**


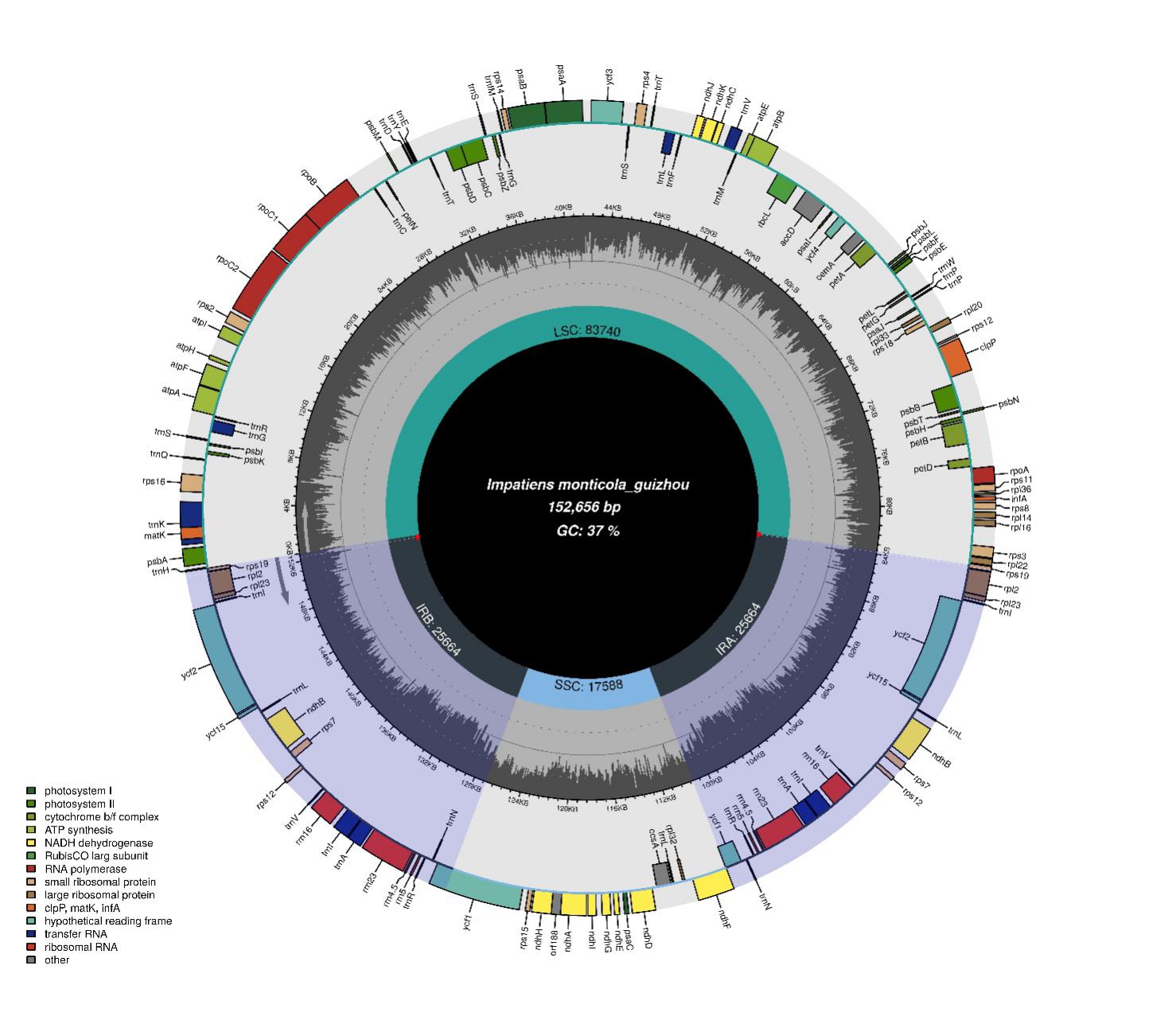


**Fig 1. Chloroplast genome structure of *Impatiens* specimens *I. monticola* from Guizhou.** The species name and specific information regarding the genome (length, GC content, and the number of genes) are depicted in the center of the plot. In the first inner circle, the optional GC content is depicted as the proportion of the shaded parts of each section. The length of the corresponding single short copy (SSC), inverted repeat (IRa and IRb), and large single-copy (LSC) regions is also given. The gradient GC content of the genome is plotted in the second circle with zero level based on the outer circle.
