# Supplementary material for: Complete chloroplast genomes of Impatiens cyanantha and Impatiens monticola: Insights into genome structures, mutational hotspots, comparative and phylogenetic analysis with its congeneric species
Source: PLoS One. 2021 Apr 2;16(4):e0248182. doi: 10.1371/journal.pone.0248182 (PMC8018631; doi:10.1371/journal.pone.0248182)

| **S2 Fig. Codon content of 20 amino acid and stop codons in all protein-coding genes of the chloroplast genomes of seven Balsaminaceae specimens.** |
| --- |


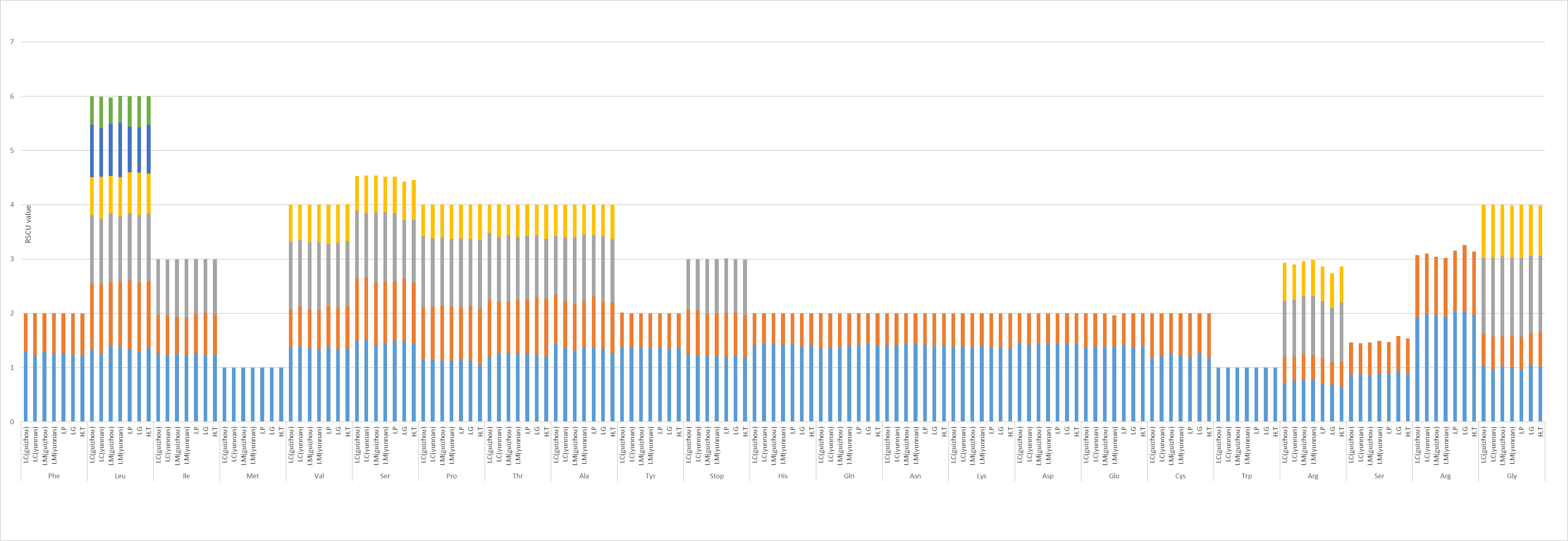

Supplement: S2 Fig — (DOCX) [file pone.0248182.s010.docx]
